# Community readiness for collecting stray dogs in Aradan County: a quantitative‐qualitative study

**DOI:** 10.1186/s13620-021-00184-4

**Published:** 2021-04-01

**Authors:** Tahereh Kamalikhah, Somayeh Mirrezaei, Tahereh Rahimi, Leila Sabzmakan, Safiye Ghobakhloo

**Affiliations:** 1 Department of Health Education and Health Promotion, Semnan University of Medical Sciences, Semnan, Iran; 2 Public Health Faculty, Iran University of Medical Sciences, Tehran, Iran; 3 Vice chancellor for Health Affairs, Qom University of Medical Sciences, Qom, Iran; 4 Department of Public Health, Jiroft University of Medical Sciences, Jiroft, Iran; 5 Department of Health Education and Health Promotion, Alborz University of Medical Sciences, Karaj, Iran; 6 Department of Environmental Health Eng, Kashan University of Medical Sciences, Kashan, Iran

**Keywords:** Stray dog, Community Readiness Model, Qualitative study

## Abstract

**Background:**

Paying more attention to free-roaming dogs’ population control seems to be necessary because of public health and environmental problems. The present study used the community readiness model to determine the readiness of Aradan County in terms of collecting stray dogs.

**Methods:**

This study is a quantitative-qualitative research study conducted in Aradan County in Semnan Province of Iran. The semi-structured questionnaire uses the six dimensions of the Community Readiness Model as guideline, with 36 items used for the interview. The interviews lasted 45 to 100 minutes with 11 key members including the governor, prefect, mayor’s assistant, city council chairman, key trustees, officials responsible for environmental health network, officials responsible for environmentalism of the city, and governors of a rural district. In quantitative part two, assessors read the interviews carefully and assigned scores based on the rating-scale form suggested by the guideline itself for scoring each dimension. A qualitative directed content analysis with deductive approach was used for analyzing the collected qualitative data.

**Results:**

The study involved 11 key members of Aradan County, all of whom were male. Most of the participants were over 40 years old and with five years of work experience (73.6 %). The mean score of each six dimensions in Aradan County were: Community efforts (4.78), Community knowledge of efforts (4.28), Leadership (4.90), Community climate (4.38), Community knowledge about the issue (4.20) and Resources related to the issue (3.29) respectively. Community readiness in Aradan County and Aradan City was generally estimated to be in the preplanning stage, whereas vague public awareness was found in the rural areas.

In the qualitative part, 870 initial open codes, 589 refund codes, 19 subcategories and 6 themes emerged, including (a) community efforts, (b) community knowledge of the efforts (c) leadership, (d) community climate, (e) community knowledge of the issue, and (f) resources related to the issue.

**Conclusions:**

For improving the process of collecting the stray dogs, it is necessary to focus on holding educational sessions for the public to increase their partnership and justify the responsible organizations’ activities to collaborate and provide the necessary financial resources.

## Background

Animal bites are a major public health problem in children and adults around the world. The health effects of animal bites depend on the health of the animal species, the health status of the bitten person, and appropriate access to different health care services in community [1]. In studies conducted between 1993 and 2013 there were 230,019 reported cases of animal bites in Iran [2]. Among the different types of animal bites, dog bites are the most important due to the transmission of various microorganisms which can cause infectious wounds, as well as the increased risk of rabies transmission [3]. Meanwhile, stray dogs are often trouble makers; it is believed that the abundance of stray dogs is associated with an increased risk of attack and biting, and zoonosis such as rabies, leptospirosis, intestinal problems, and leishmaniasis (especially in poor areas) [4]. The rabies case-fatality rate in people approaches 100 %, yet it is completely preventable [5]. Also, over ten million people bitten by animals in different parts of the world receive anti-rabies treatment each year to prevent rabies. However, more than 55,000 people die from rabies, 99 % of whom live in developing countries, largely due to insufficient control of rabies [6]. Annually, 30,000 deaths occur in Asia, but the painful reality is that among the deaths caused by rabies in humans, 15 % of deaths occur among children under 15 years of age [5]. The high mortality rate of rabies in many developing countries indicates that despite the existence of effective rabies vaccines for humans and animals, the preventive and control actions taken in these contexts are insufficient [7]. In this regard, eliminating stray dogs and other animals by shooting and poisoning is still practiced in some countries; however, this has the least effect on the transmission of rabies [3]. For these reasons, the illegal killing of dogs as a tool to control rabies has now been condemned worldwide. Currently, the reduction of dogs in some environments has been replaced by a variety of humanitarian approaches that aim to have lasting and positive effects on the population of dogs and the communities in which they live. Professional tools such as vaccination and other methods of disease control, food access control (habitat control), Domestic Dogs and Responsible Dog Ownership (RDO) promotion, reproductive prevention and control, identification and registration of individual dogs, access to shelters, return to home centers, care facilities, and the approval of law can collectively contribute to more effective programs [8].

According to some scientific findings, community-based monitoring systems for animal biting and rabies control and management have been successful and cost-effective in other areas [9, 10]. Before starting community-based intervention programs, the community’s readiness for change and the capability of communities to address the issue must be identified.

Community readiness is defined as the observable and psychological characteristics of a community affecting the society’s ability to initiate change [11].

The present study uses the Community Readiness Model to determine the readiness in Aradan to collect stray dogs. The Community Readiness Model is a model for community change that combines culture, resources, and the stage of community readiness to address an issue in particular. This model has six dimensions, including key factors that highlight a society’s readiness to act on an issue. These six dimensions include community efforts, community knowledge of efforts, leadership, community climate, community knowledge about the issue, and resources related to the issue. The situation of the community on each of these dimensions is the overall basis for the community readiness stage. The general stages of community readiness include 9 steps: (1) No awareness (issue is not identified as a problem by community members); (2) Denial/Resistance (issue recognized by a few members of the community, but the overall community belief is not accompanied by addressing this problem); (3) Vague awareness (some people believe that there is a problem to be addressed, but they do not have an immediate motivation to start changes); (4) Preplanning (some community members and leaders believe a problem exists and actions should be considered); (5) Preparation (active planning to address the issue has been done with the participation of the community members); (6) Initiation (activities and programs are implemented); (7) Stabilization (programs and policies are running and stable); (8) Confirmation/Expansion (community members value the implementation of programs and policies and decision makers support them); and (9) A high level of community ownership (program evaluation is done) [12, 13].

Currently, studies on the context of society are needed to take more fundamental steps and actions. Therefore, considering the global importance of the issue as well as the importance of planning for preventive interventions, the present study was conducted to investigate the community readiness for collecting stray dogs in Aradan County using the community readiness model.

## Methods and materials

### Study design and participants

This study is a quantitative-qualitative research conducted in Aradan County in Semnan Province, Iran in 2017. The study population consisted of all the authorities and trustees of Aradan County, including governor, prefect, mayor assistant, city council chairman, key trustees, officials responsible for the environmental health system or network, officials responsible for the environmentalism of the city, governors of a rural district, police, and the officials in the Department of Agriculture.

### Data collection

For the purpose of this study, the Community Readiness Model was used. For this purpose, the guideline and booklet of the model [14] was translated into Persian [15]. To determine the level of community readiness, a semi-structured questionnaire was used to collect data about 36 items on the six dimensions of community model including (a) community efforts (What are the efforts, programs and policies that address collecting story dogs?), (b) community knowledge of efforts (to what extent are community members aware of collecting story dogs efforts and are these efforts available to all sections of community?), (c) leadership (To what extent do community leaders support collecting story dogs?), (d) community climate (what is the prevailing attitude of the community about collecting stray dogs?), (e) community knowledge about the issue (how much do community members know about collecting stray dogs, their consequences and their effects?), and (f) resources related to the issue (to what extent are local resources including people, time, money and space available to support the efforts about collecting stray dogs?). On average, there were six questions for each dimension, with some follow-up questions. These questions were asked by two assessors from key informants and provide an adequate insight into collecting stray dog problems. Each interview took 45 to 100 minutes with 11 key members of the targeted community to achieve data saturation in qualitative part.

Selection of participants was performed following an official request to the governorate, and our introduction to the county health work group was arranged by the governorate. Meanwhile, some organizations were reluctant to cooperate and did not give time for interviews. After obtaining consent and permission from each participating individual, the digital recording of the interview was performed (two of the interviewees did not allow recording. In these two instances, careful notes were taken instead) while recording brief notes of their non-verbal observations and behaviors.

### Data analysis

As for the scoring method in the quantitative part, after the interviews two assessors read the interviews carefully and outlined the terms related to their scoring. Then, the rating scale form developed and suggested by the guideline itself for the scoring of each dimension was observed [14]. That is, for each dimension 9 items are assigned, for example, the first degree of (a) ‘community efforts’ dimension is the lack of knowledge, the second degree of this dimension is lack of actions in the society about this subject. To achieve the score of a particular stage, the score of all previous stages must be achieved. On the scoring form, each assessor entered his or her own score in the personal scoring table. When the independent scoring was completed, the two assessors met each other and discussed their scores to reach a consensus on the scores, then the composite scoreboard was completed. The scores in each row were summed to obtain the overall score for each dimension. To find the calculated score for each dimension, the overall score was then divided by the number of interviewees. The assessors ranging from 1 to 9 were compared to the specified table to determine the community readiness stage

**Table 1:** Scores for each stage of community readiness

Stages of model	Score
No awareness	1
Denial / resistance	2
Vague awareness	3
Preplanning	4
Preparation	5
Initiation	6
Stabilization	7
Confirmation / expansion	8
High level of community Ownership	9

A qualitative directed content analysis with deductive approach was done based on Graneheim and Lundman’s methods in the qualitative part [16]. In order to reach a general perception, the transcript of the recorded interviews was prepared on the same day and reviewed several times. Based on the concepts behind each word and sentence, the semantic units were examined, summarized and then converted into codes. The different codes were compared according to their similarities and differences and were allocated into subcategories; they were placed under six themes based on the six dimensions of community readiness model.

Guba and Lincoln’s precision criteria in qualitative research were used to ensure the reliability and accuracy of the data [17]. Credibility was achieved by the prolonged engagement of the researcher with the participants of the study and allocating enough time for data collection. Conformability was assured by observing revision, while dependability was examined through the confirmation of the extracted codes to other participants. Also, the consistency of the findings was assured by transcribing and interpreting the information collected immediately after the interview and by offering the transcripts of the interviews and the extracted codes to some participants and verifying of them). Finally, transferability (i.e. describing and documenting the data) was achieved by writing the research process and using a sampling technique to maximize diversity among the study population.

## Results

The study involved 11 key members of Aradan County, all of whom were male. 92 % of them had university education, while 73.6 % of them were over 40 years old with at least five years of work experience.

The mean score for all six dimensions was 4.30 out of 9 points. The overall mean community readiness in Aradan County, Aradan City and the surrounding villages were 4.30, 4.50 and 3.52, respectively. Table 2 shows the mean score of evaluation for all six dimensions. In total, resource dimension had the lowest (3.29) and leadership dimension (4.90) the highest score.

**Table 2:** Mean score of assessment from each dimension in the city and the village

Dimension	Assessor 1 (County)	Assessor 2 (County)	Combined (County)	Urban (Combined)	Village (Combined)
Community efforts	4.93	4.63	4.78	5.02	3.81
Community knowledge of efforts	4.57	4	4.28	4.48	3.50
Leadership	5.05	4.76	4.90	5.27	3.43
Community climate	4.35	4.40	4.38	4.55	3.70
Community knowledge about the issue	3.95	4.45	4.20	4.26	3.93
Resources related to the issue	3.21	3.38	3.29	3.42	2.78
Total	4.34	4.27	4.30	4.50	3.52

According to the guideline, in order to calculate the community readiness stage, the total mean of the assigned scores is rounded down and the decimals have no effect on the community stage. After comparing the scores obtained for Table 1, community readiness in Aradan County and Aradan City were generally both estimated to be at the stage of “Preplanning”, whereas “Vague awareness” was found in rural areas.

In the qualitative part, the 6 themes and 19 subcategories generally emerged from 870 initial open codes (Table 3).

**Table 3:** Themes, Subcategories and open codes

Themes	Subcategories	Examples of Open Codes
Community efforts	Killing stray dogs in the past Collecting but not killing the stray dogs now Organizational activities The difference in developing the project between the city and the village	The use of poisons around waste is an old problem-solving method Sterilize dogs as a new method Start of activities by forming a stray dog
Community knowledge of the efforts	Governmental actions to provide information Public actions to get information People’s relative awareness of efforts	Informing the community members by the municipality with installing banners before implementing the plan People go to healthcare centers in case of animal bites for more information
Leadership	Supervisory organizations Municipality and government officials in the village (‘Dehyari’) mostly responsible for this issue The relative leadership of other organizations	The municipality, the governorate and the health sector as the leaders of this issue in the community The role of religious leaders and other organizations in creating a culture on the subject of stray dogs
Community climate	Positive attitude and public demand for implementing the project Negative view of Society for Protection against Cruelty to Animals (SPCA) about how stray dogs are collected	Public attention at the time of project implementation The problem of stray dogs in the villages is more serious and the councils are more aware of this issue
Community knowledge of the issue	In the city: People’s relative awareness of the dangers of stray dogs and the related diseases Lack of people’s awareness of the effective actions Public awareness of the responsible organizations. In the village: Lack of villagers’ awareness of the dangers of stray dogs and the related diseases Public awareness of the responsible organizations	In the city: Lack of public awareness about dog contamination Low public awareness about keeping a pet In the village: Lack of awareness due to the low susceptibility of the villagers to stray dogs
Resources related to the issue	Differences in resources in urban and village, opportunities and challenges	Lack of funds in the discussion of veterinary education Volunteer to implement the project Lack of plans to expand the project

- **A) Community efforts**: The interviewees described past efforts in the community to include collecting stray dogs through aggressive methods such as baiting and using weapons. Some of these methods were believed to have strengths including the use of popular volunteers as hunters and the fact that 95 % of stray dogs were collected from the community. For example, one of the interviewees mentioned, “*In the past, when collecting was done with weapons, people were 100 % satisfied and some were even volunteers, but now there is no plan for keeping the collected dogs that are collected alive and left a few kilometers away perhaps leading to problem in other cities or in some cases they come back to city again*”. However, interviewees reported that the previous method would kill other animals due to the use of poison for baiting and that they were opposed by SPCA. The participants and authorities described current community efforts using contractors during the various stages of collecting dogs alive followed by sterilization, vaccination, and release. Current efforts in the countryside and villages were highly dispersed, and the villagers themselves point out that we do not know if we are really responsible, and if it is so, why we do not have the funds for it, and also one of them accused the health system for this problem. One of the governors of the villages stated: *“The only thing we can do when it comes to people’s complaints is to write a letter to the healthcare system and the prefect; the police don’t involve themselves at all. We have one or two alleys in the village, believe me, there are maybe more than 12 or 30 families living up the alley…. about 12 dogs come here and people can’t sleep here at night or during the day, the dogs jump on motorcyclists and they fall down”.* Other efforts in this community included organizational activities, including a county-level destruction committee headed by the governor, which would occasionally address this issue when it becomes a problem.
- **B) Community knowledge of the efforts**: The community was somewhat involved due to the problems caused to them by the stray dogs and they were being informed by installing banners. Before the project was implemented at the city-wide and village-wide levels, there were no serious actions and no public awareness. In this regard, a representative of the municipality stated, *“Twenty days before the project is implemented, we will install banners and announce that those dogs with collars and owners should be kept by their owners during the project so that the following day they won’t claim that you’ve arrested our dogs”.* It was also stated that people were more aware of the efforts after the project was implemented although people were not aware of how the project was being carried out in detail. However, generally they knew that actions were being taken. According to officials, people also sought information and awareness through the healthcare system, municipality, and the internet. Concerning the efforts and the practitioners involved, officials noted that the townspeople had more awareness in comparison to people from the villages.
- **C) Leadership**: With respect to the leadership dimension in the model, responsible authorities overwhelmingly recognized this as an inherent duty of the municipality and the authorities of villages, and they also recognized some organizations as having a supervisory role such as governors and prefects. The governors stated that the governorate is in charge of monitoring and coordinating the organizations and is trying to control the situation through the establishment of a food safety and health group work when there is a crisis in the community. On the other hand, the governors of the villages believed that the prefect had very little involvement, with only some limited correspondence. In this regard, one of the governors of the villagers declared, *“The district administration once gave us a letter saying that whatever else was done a few years ago, it would be our job to prioritize it according to the needs of the village. For example, you announce this has happened to me and dogs constantly bother us, we’ll then start our work and take care of it based on your request”.* It was also stated that they do not know what to do now; *“we must either destroy dogs by ourselves or must start letter writing, which means making a bungle of the case because no police force will be involved”.* Other organizations in the project also had a leadership role. Some of them supported the project by participating in committee meetings or sending a thank-you note to the municipality for doing so. The national leaders participated as national and provincial working groups to set guidelines, whereas the religious leaders participated in the project through acculturalization.

*“In the context that we collected the dogs, many people even offered thanks during Friday Prayer (special prayer for Muslims)”. Mr. [….] Thanked this municipality action”.*

**D) Community climate**: Generally, Ardan people have a positive view of collecting stray dogs as evidenced by their repeated requests to implement this project. Furthermore, after the project had been carried out, people expressed their satisfaction with the project. The troubles that the dogs caused for people included the injuries of women and children while being chased by dogs, biting people, terrifying people at night by barking, preventing people from being able to walk in the city, rushing to trash bins and scattering the trash, and making an inappropriate view of the city for guests from other cities which was especially unattractive to guests when mating in herds during the breeding season. Other troubles included damage to alfalfa, wheat and barley fields, especially in the village, accidents of motorcyclists with dogs, transmission of infections and diseases, etc. In this regard, one of the trustees mentioned, *“I had planted vegetables on the ground some time ago, but the herd dogs squished the greens and vegetables.”*

On the other hand, there was some kind of fear and concern among the authorities who suggested that caution should be exercised in collecting the dogs because if, for example, the old method is used, it would be filmed by the Society for Protection against Cruelty to Animals, or by other (ordinary) people, and would become widespread on the internet. This would cause anger and confusion by people across the country viewing the video online.

**E) Community knowledge about the issue**: People in the city were relatively aware of the issues and problems such as diseases that can be caused by dogs and animal bites. In addition, the townspeople were aware of the organization responsible and to whom they should refer any injury or complaint to. In the villages however, people were not aware of the dangers of stray dogs and related diseases. One of the governors of the villagers mentioned, *“People have little knowledge and the reason the dogs were raised so much was because of the thefts. Now that thefts have declined, people still have that thinking although villages don’t really care much about stray dogs because they are parts of their lives. Honestly speaking, the notification of announcements was poor; most people are ranchers and farmers and have a dog themselves. If there is a stray dog nearby, they aren’t afraid. They run after the dog with a stone, but the situation in the town is the other way around”.* According to the governors of the villagers, the villagers are not aware of effective actions and of the law on stray dogs, but they are aware of the responsible authority, which is the healthcare system and us. *“They don’t know. They only know that if the dog attacks them and catches their legs or hands or breaks them, they should report this to us or the healthcare system and take the injured people to the police station nearby…. That’s it”.*

**F) Resources related to the issue**: The resources available for this project were provided by the municipality, which paid the contractor 800000 IRR (20 Euro) per dog. But in the villages there was no funding available for this issue, and the governors of the villages run dogs themselves. In this regard, a city council official stated, *“We have determined for municipalities to contract with the contractor for each dog roughly* 800000 IRR (20 Euro); *we do not need any other resources or help of charity. This amount of money and this plan is enough for this purpose.”*

## Discussion

The aim of this study was to assess the situation of Aradan City in the field of collecting stray dogs using the Community Readiness Model. According to the results of this study, the overall stage of community readiness in Aradan County was in the pre-planning stage. There were notable differences between the level of community readiness in the city versus the level of readiness in the villages. The stage of community readiness in the city was in the pre-planning stage, while it was in the stage of vague awareness in the villages. It can be concluded that the stage of community readiness of Aradan in the field of collecting stray dogs was low, which was much more of a problem in the villages than in the city. One study conducted in Yucatan found that 63 % of dogs roam without supervision and are fed by litter, especially in rural areas [18]. In our study, leadership dimension scored highest and resource dimension lowest. In the study of Sliwa et al., where the Community Readiness Model was applied to obesity prevention, the results showed that the stage of community readiness was in the preplanning stage, in which community climate dimension had the lowest score and the leadership dimension had the highest score [19].

From the community efforts dimension, some actions were taken in the past, especially in the city to collect stray dogs, such as collecting stray dogs using aggressive methods including hunting and using arrows, which have had positive results for control. However, due to new national legislation it could not be implemented anymore, and current efforts include setting up a committee at the governorate level and convening meetings in times of trouble and collecting alive stray dog from time to time without a proper plan and without providing space to keep dogs so that the released dogs would return to the city. Therefore, the community readiness of the society has been placed at the pre-planning stage, and this shows that it is not enough to take action. Yet, it must be planned further, and continuous and periodic actions should be taken to achieve the desired results. In this model, the preplanning stage refers to a stage when it is clearly recognized that something needs to be done, and also that a group is working on it, although the efforts are not focused or detailed yet [14]. Cortez-Aguirre et al. found that similar to other developing countries, no actions were taken to control dogs, and there were no animal control centers, permanent sterilization campaigns, or public information for the responsible dog care centers in Campeche City of Mexico [4]. In this study, the community efforts in the villages were very vague, and even those involved were vaguely aware of the laws, mainly due to the lack of negative attitudes toward dogs and people’s needs for dogs as well as the lack of funding for action. Vague awareness means that the society has recognized that this is a local problem, but they have no incentive to do anything about it. There is no clear leadership or a leader with energy or motivation to address this issue [14].

In the case of people’s awareness of the efforts, the officials also acknowledged that the people of the city are more aware than the people of the village, which was due to the implementation of the plan in the cities and the installation of banners in the streets before the implementation of the plan. People in the city considered the municipality and in the village considered government officials in the village (‘dehyari’) as responsible institutions and referred to them in case of any problems. In terms of people’s awareness of the issue, according to the officials, people in the villages were less aware than in the cities about the diseases transmitted by dogs, especially rabies. In fact, the villagers did not consider the issue of stray dogs a problem. This shows that there is a gap in knowledge in the society. In particular, health officials say that they are more focused on chronic diseases and are holding classes and educational content on healthy living. Without awareness, the society cannot be expected to be sufficiently prepared to participate in the project. Therefore, it is necessary to design and implement coherent and planned trainings with the cooperation of related organizations, especially for dog owners.

The role of appropriate educational media in this regard should not be overlooked. In this way, people’s sensitivity to the dangers of stray dogs can be increased. Unfortunately, in Iran, there is not enough information in the field of public awareness about rabies, and studies have focused more on the epidemiology of this problem. In the study of Hamidzadeh Arbabi et al., the results showed that people’s awareness of rabies is low [20]. In a study conducted in Ethiopia by Yalemebrat et al., most respondents were unaware of rabies and did not know exactly how rabies was transmitted [21]. However, in a study in India [22], most respondents (74.1 %) were aware of rabies. In another study by Sambo [7], the most common source of information about rabies (70 %) comes from neighbors, parents and friends, as well as 15 % through mass media, 12 % through professionals such as health workers, researchers or school teachers, and only 3 % from other resources such as pamphlets. In the Ethiopian study [21], most respondents in the study stated that the main place of learning was the workplace.

In terms of leadership, all participants considered the municipality and the government officials in the village (‘dehyari’) to be the main responsible institutions, and the governorate and prefect organization played a supervisory role. It seems that the organizations should participate more in the implementation of the project. On the other hand, according to the officials, one of the main reasons that prevented the proper and complete implementation of the stray dog ​​collection project was the low budget, which prevented the project from being carried out, especially in the villages where it is necessary that health policymakers provide the necessary conditions for justice in the supply and distribution of resources. Given the fact that these villages favor the supervision of prefect, prefects should be responsible for monitoring, coordinating and forming a health working group with the aim of overseeing the collection of dogs with help of the governors of the villages. On the other hand, there was disruption and confusion and the main responsible organization was not clear to the governors of the villages. For example, one of the governors of the villages considered the health center responsible for collecting stray dogs, which indicates that the authorities are not aware of the rules for collecting stray dogs. In the Cortez-Aguirre et al. study, it was noted that the lack of government action could increase stray dogs in different areas [4].

In a study carried out in India, only 33.5 % of respondents stated that people had no role in controlling the population of stray dogs and believed the government was largely responsible for controlling stray dogs [22]. In terms of community climate dimension, the people of Aradan had a positive view on the issue of collecting stray dogs although in the villages this issue was not very important. The general atmosphere of the Aradan community is in favor of collecting stray dogs although according to officials their participation in the collecting stray dog plan was low because the people considered the responsibilities in this regard to be the responsibility of the municipality and the village council. As mentioned earlier, it is recommended to implement training programs for dog owners to justify vaccination, not to release dogs, especially old and sick dogs and other sections of the society. This study is inconsistent with Matibag et al. study in Sri Lanka [23] in which most participants would feel responsible for the increase in the population of stray dogs and did not believe that responsibility should simply be delegated to the authorities.

Given the negative view of SPCA about the wrong way to collect stray dogs, it seems that the presence of a representative of this association in the working group meetings can be fruitful. Also, the role of these associations in influencing the atmosphere of the society in relation to people’s participation in collecting stray dogs should not be ignored. One of the limitations of this study, which can be considered as a limitation of the Community Readiness Model, was the lack of interviews with the people of the city. This was not mentioned in the model, and only the interviews with the leaders and trustees of the people were recommended. To ensure that information about the broader community atmosphere is captured, and community readiness is assessed, it is recommended that interviews are conducted with groups of people, especially dog ​​owners, was the best action to be taken.

In this study, key informants suggested intervention strategies in Aradan County should include coordination between organizations to gain full support for funding, setting precise tasks for each organization, and thus careful planning and implementation, allocating space for keeping dogs, sterilizing them or even in some cases breeding and selling them to organizations such as fire stations and generating income in addition to providing more information. Also guideline emphasize that when community readiness is in pre-planning stage, information through lecture or media should be provided to the public, leaders must meet and speak with them about reasons for the problem, existing effort must be reviewed and focus group discussion about issues and the design of appropriate intervention strategies is suggested. In the villages, it is possible to increase public awareness of these efforts through individual contacts and media programs that must be chosen carefully and prepared based on the community readiness stage to justify that in many cases there is no need for an individual to have a dog. Thus, dog owners need to take responsibility for the dogs and not release them when they get older. Allocating more budgets and clarifying the role of the governors of the villagers can also help in the villages. As per the guideline [14], when community readiness is in the vague stage, it is recommended that information is disseminated at local occasions and ceremonies and also through advertisements, posters and billboards, articles in local newspapers and via informal interviews with people. Due to the mortality of the rabies, a large amount of money is allocated annually by the Ministry of Health to provide vaccines and serums for the treatment and prevention of people who suffer from animal bites. This contributes to the achievement of an appropriate budget for dog prevention, which is based on prevention. It is recommended that future intervention strategies should be based on the results of studies such as this.

## Conclusions

Community readiness in Aradan County was estimated to be at the preplanning stage, whereas vague public awareness was found in rural areas. For improving the process of collecting the stray dogs, it is necessary to focus on holding educational sessions for the public to increase their partnership and justify the actions of responsible organizations to collaborate and provide the necessary financial resources.

## Data Availability

The dataset supporting the conclusions of this article are not publicly available due to confidentiality but are available from the corresponding author on reasonable request.

## Abbreviations

RDO: Responsible Dog Ownership
SPCA: Society for Protection against Cruelty to Animals

## References

[1] Shamshirgaran SM, Barzkar H, Ghaffari-Fam S, Kosha A, Sarbakhsh P, Ghasemzadeh P (2017). Epidemiological characteristics and trends in the incidence of animal bites in Maku County, Islamic Republic of Iran, 2003 – 2012. East Mediterr Health J.

[2] Abedi M, Doosti-Irani A, Jahanbakhsh F, Sahebkar A (2019). Epidemiology of animal bite in Iran during a 20-year period (1993–2013): A meta-analysis. Trop Med Health.

[3] Seimenis A (2008). The rabies situation in the Middle East. Dev Biol (Basel).

[4] Cortez-Aguirre GR, Jiménez-Coello M, Gutiérrez-Blanco E, Ortega-Pacheco A (2018). Stray dog population in a city of southern Mexico and its impact on the contamination of public areas. Vet Med Int.

[5] Singh R, Singh KP, Cherian S, Saminathan M, Kapoor S, Manjunatha Reddy G (2017). Rabies–epidemiology, pathogenesis, public health concerns and advances in diagnosis and control: a comprehensive review. Vet Quart.

[6] Esmaeilzadeh F, Hatam N, Esmaeilzadeh Z, Rajabi A, Anami M, Vahedi S (2014). Effectiveness of post-exposure rabies prophylaxis. Tehran Univ Med J.

[7] Sambo M, Lembo T, Cleaveland S, Ferguson HM, Sikana L, Simon C, et al. Knowledge, attitudes and practices (KAP) about rabies prevention and control: a community survey in Tanzania. PLoS Neglect Trop Dis. 2014;8(12). 10.1371/journal.pntd.0003310 PMC4256472 25473834

[8] Taylor LH, Wallace RM, Balaram D, Lindenmayer JM, Eckery DC, Mutonono-Watkiss B (2017). The role of dog population management in rabies elimination-a review of current approaches and future opportunities. Front Vet Sci.

[9] Dodet B, Goswami A, Gunasekera A, de Guzman F, Jamali S, Montalban C (2008). Rabies awareness in eight Asian countries. Vaccine.

[10] WHO Technical Report Series. WHO Expert consultation on rabies: First report. Geneva: Switzerland. 2005:931. 16485446

[11] Wallerstein NB, Duran B (2006). Using community-based participatory research to address health disparities. Health Promot Pract.

[12] Chilenski SM, Greenberg MT, Feinberg ME (2007). Community readiness as a multidimensional construct. J Commun Psychol.

[13] York NL, Hahn EJ (2007). The Community Readiness Model: evaluating local smoke-free policy development. Policy Polit Nurs Pract.

[14] Plested BA, Edwards RW, Jumper-Thurman P. Community readiness: A handbook for successful change: Tri-Ethnic Center for Prevention Research: Colorado State University; 2006.

[15] Karimi M, Kamalikhah T (2018). Community Readiness: A handbook for successful change.

[16] Graneheim UH, Lundman B (2004). Qualitative content analysis in nursing research: concepts, procedures and measures to achieve trustworthiness. Nurs Edu today.

[17] Hsieh H-F, Shannon SE (2005). Three approaches to qualitative content analysis. Qual Health Res.

[18] Ortega-Pacheco A, Rodriguez-Buenfil JC, Bolio-Gonzalez ME, Sauri-Arceo CH, Jiménez-Coello M, Forsberg CL (2007). A survey of dog populations in urban and rural areas of Yucatan. Mexico Anthrozoös.

[19] Sliwa S, Goldberg JP, Clark V, Collins J, Edwards R, Hyatt RR, Junot B, Nahar E, Nelson ME, Tovar A, Economos CD (2011). Using the Community Readiness Model to select communities for a community-wide obesity prevention intervention. Prev Chronic Dis.

[20] Hamidzadeh Arbabi Y, Rezakhani H, Savadpoure M, Nakhostine B, Haji Gahramani M, Babai Y (2013). Impact of health education on incidence of animal bites and knowledge on rabies and preventive behaviors in selected villages of Ardabil city. J Health.

[21] Yalemebrat N, Bekele T, Melaku M (2016). Assessment of public knowledge, attitude and practices towards rabies in Debark Woreda, North Gondar, Ethiopia. J of Vet Med Anim Health.

[22] Herbert M, Basha R, Thangaraj S (2012). Community perception regarding rabies prevention and stray dog control in urban slums in India. J Infect Public Health.

[23] Matibag GC, Kamigaki T, Kumarasiri PV, Wijewardana TG, Kalupahana AW, Dissanayake DA (2007). Knowledge, attitudes, and practices survey of rabies in a community in Sri Lanka. Environ health Prev Medicine.

